# Supplementary material for: Interpreting generative adversarial networks to infer natural selection from genetic data
Source: Genetics. 2024 Feb 22;226(4):iyae024. doi: 10.1093/genetics/iyae024 (PMC10990424; doi:10.1093/genetics/iyae024)
Supplement: iyae024_Supplementary_Data [file iyae024_supplementary_data.zip › Supplemental_Figures_GENETICS-2023-306637.pdf]

Supplementary Material

Interpreting Generative Adversarial Networks to Infer Natural Selection from Genetic Data

Rebecca Riley[1], Iain Mathieson[2], and Sara Mathieson[1,†]

[1] Department of Computer Science, Haverford College, Haverford, PA
[2] Department of Genetics, University of Pennsylvania, Philadelphia, PA
[†] Corresponding author: Sara Mathieson, smathieson@haverford.edu

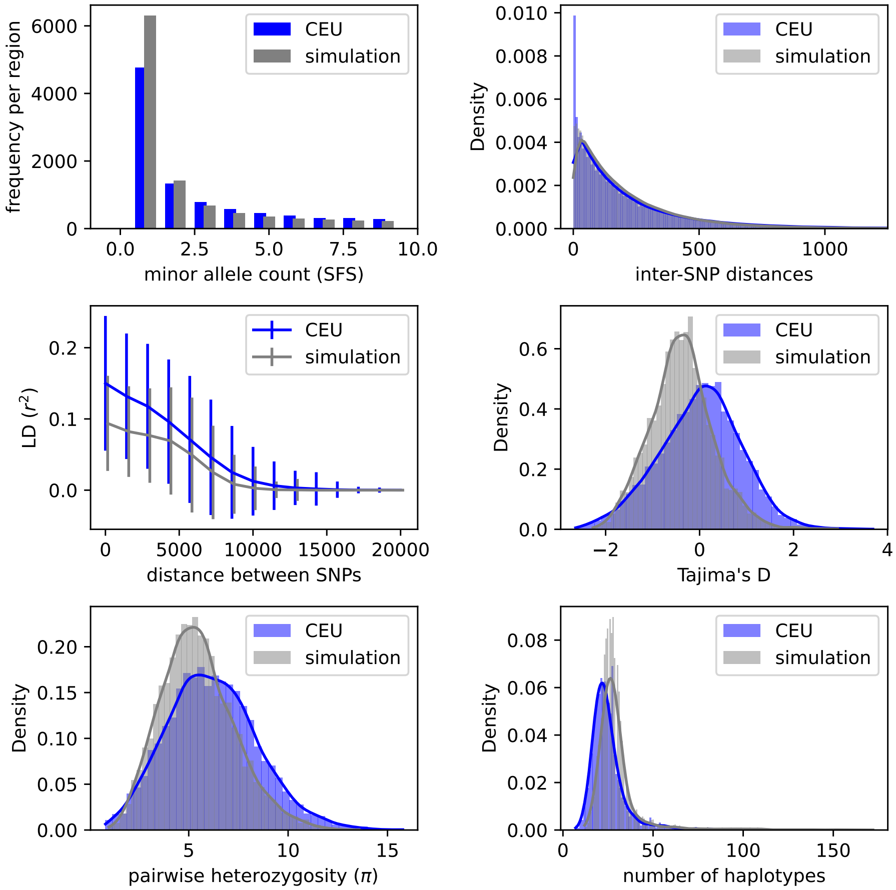

Figure S1: Summary statistic comparison for a failed pg-gan training run. In this example (seed 12), CEU was used as the training data and the learning process failed (see Figure S2 below). Typically with failed training, the generator takes a random walk around the parameter space, since the discriminator essentially ignores the input data (whether real or simulated). In most cases this results in an inferred demographic model that fails to recapitulate properties of the real data, which is what we see in these summary statistics.

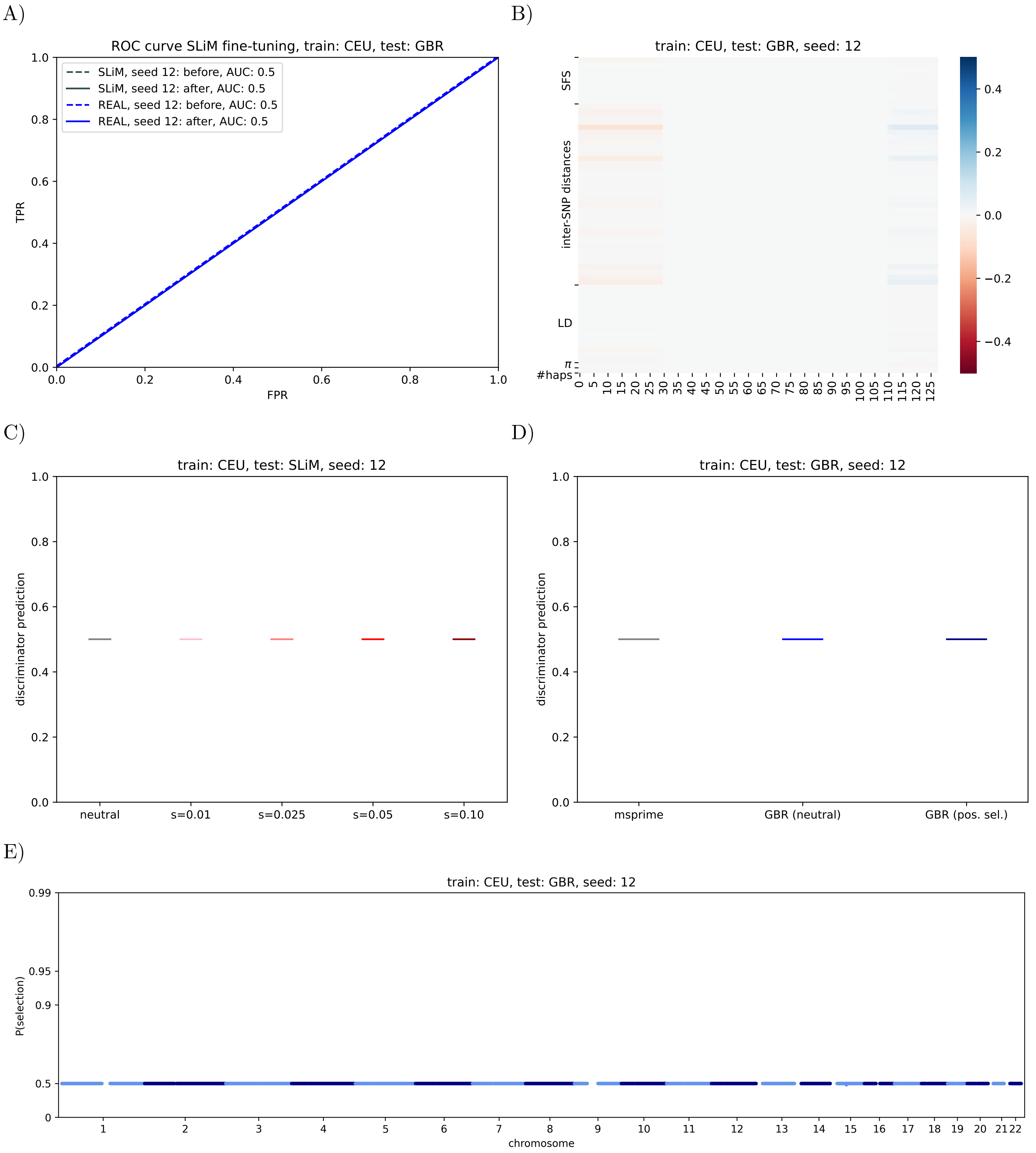

Figure S2: Example of a CEU-trained discriminator with test data from GBR (seed 12). Ten out of the 60 training runs of `pg-gan` failed and show this general pattern, where the discriminator predicts the same value for all regions (essentially ignoring the input data). A) Fine-tuning with selection simulations did not change the random guessing pattern. B) In our interpretability analysis there is little correlation between discriminator hidden units and any known summary statistics. Thus for the violin plots (C,D), all predictions are the same regardless of the selection coefficient or simulated/real status of the region. E) No regions are outliers in terms of their selection probabilities.

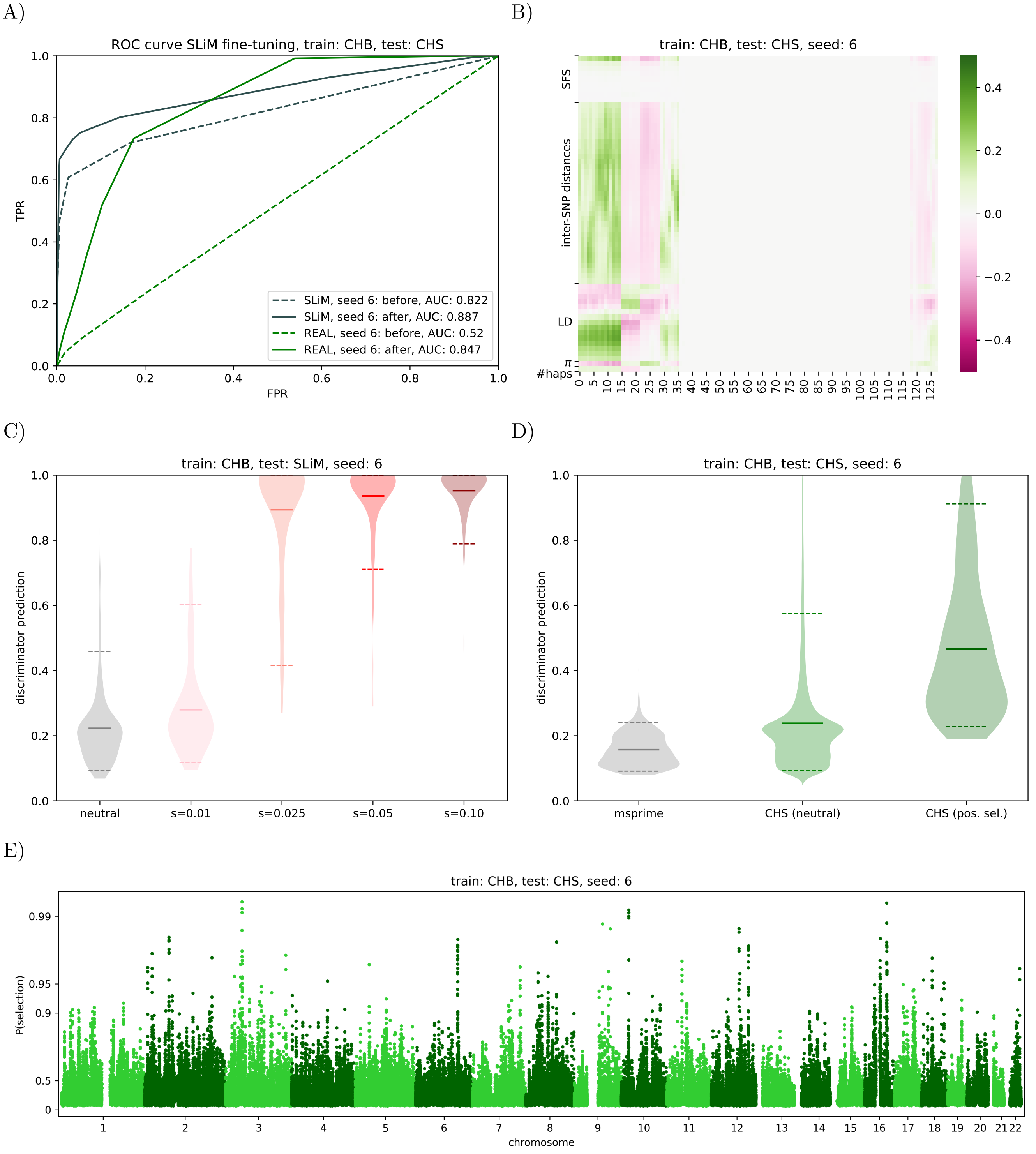

Figure S3: CHB-trained discriminator (seed 9), fine-tuning with positive selection simulations, test data from CHS. A) ROC curve showing performance on selected regions before and after fine-tuning. All predictions (both simulated and real) are made for unseen (test) data. SLiM indicates simulated data with various selection coefficients and REAL indicates selected regions from Grossman *et al* (2013). B) Correlation heatmap between discriminator hidden units (x-axis) and classical population genetics summary statistics (y-axis). The columns (hidden units) were clustered according to their similarity in terms of summary statistic correlation profiles. C) Performance of discriminator on unseen simulated data under various selection strengths. D) Performance of discriminator on selected regions from Grossman *et al* (2013). E) Genome-wide Manhattan plots of discriminator predictions on real test data.

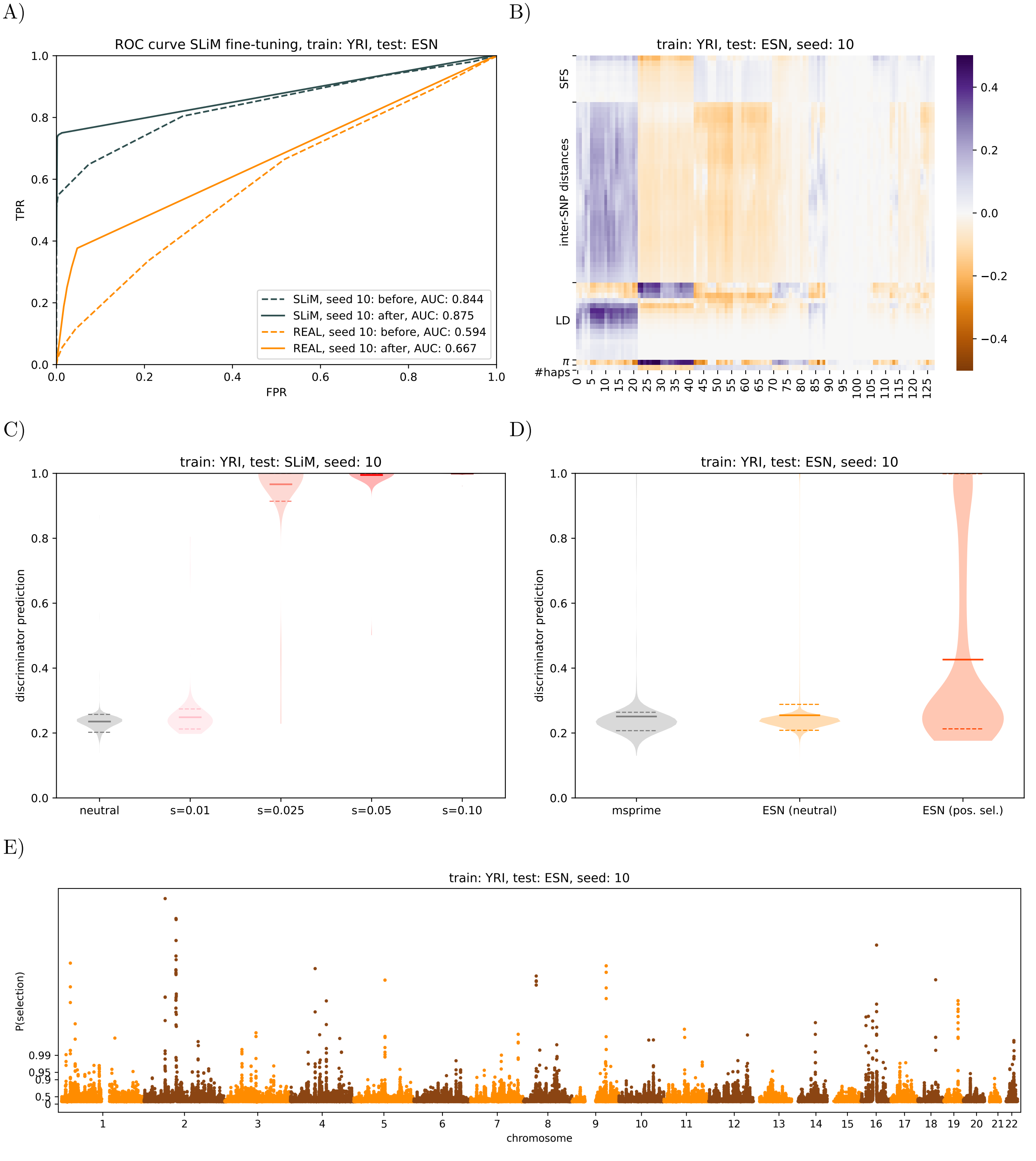

Figure S4: YRI-trained discriminator (seed 4), fine-tuning with positive selection simulations, test data from ESN. A) ROC curve showing performance on selected regions before and after fine-tuning. All predictions (both simulated and real) are made for unseen (test) data. SLiM indicates simulated data with various selection coefficients and REAL indicates selected regions from Grossman *et al* (2013). B) Correlation heatmap between discriminator hidden units (x-axis) and classical population genetics summary statistics (y-axis). The columns (hidden units) were clustered according to their similarity in terms of summary statistic correlation profiles. C) Performance of discriminator on unseen simulated data under various selection strengths. D) Performance of discriminator on selected regions from Grossman *et al* (2013). E) Genome-wide Manhattan plots of discriminator predictions on real test data.

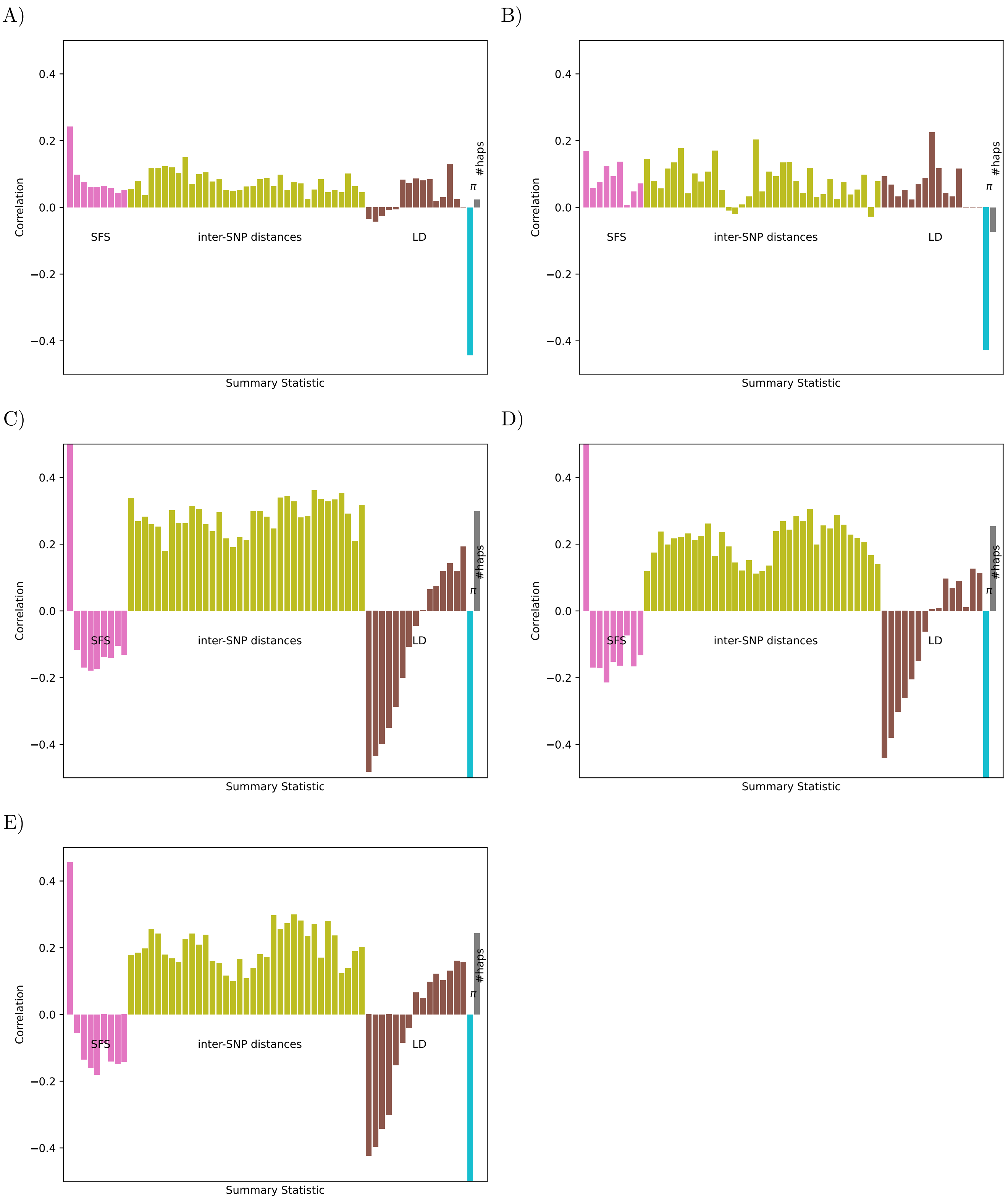

Figure S5: Correlations between summary statistics and the final discriminator prediction (for the positive selection case). The data shown here is for a CEU-trained discriminator (seed 19, as in the main text) with simulated test data (from `SLiM`). We compute the correlations between various summary statistics and the probability of selection, for different selection strengths. A) neutral data, B) $s = 0.01$, C) $s = 0.025$, D) $s = 0.05$, and E) $s = 0.1$. We can see that for selection coefficients greater than 0.01, a pattern emerges with strong correlations between the networks output and the number of singletons and π. LD statistics are also correlated, with LD for closer pairs of SNPs (left) negatively correlated and LD for further pairs of SNPs (right) positively correlated.

A)

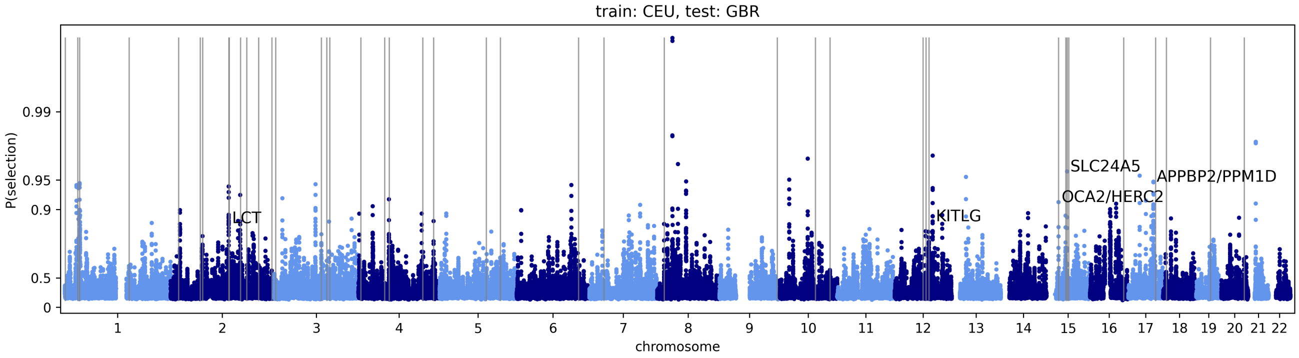

B)

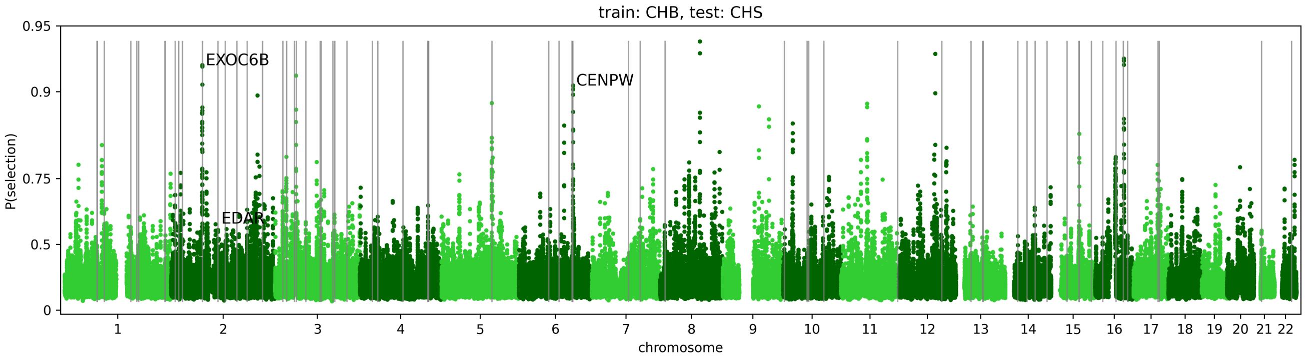

C)

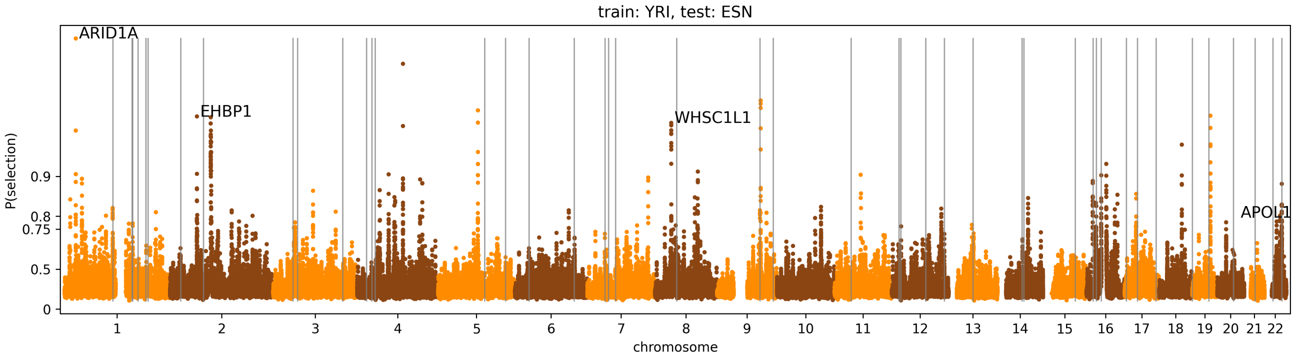

Figure S6: Ensemble results for positive selection, with known selected regions highlighted. Genome-wide selections scans for CEU/GBR (A), CHB/CHS (B), and YRI/ESN (C). In each case, the x-axis represents genomic position, the y-axis represents the probability of selection (plotted on a log scale), each point represents the average of five consecutive 36-SNP windows, and grey vertical lines represent known selected regions from Grossman *et al* (2013). In blue text we highlight regions with P(selection) above 0.75 that also overlap with these known selected regions.
